# Curing and Molecular Dynamics Simulation of MXene/Phenolic Epoxy Composites with Different Amine Curing Agent Systems

**DOI:** 10.3390/nano12132249

**Published:** 2022-06-30

**Authors:** Rui Cai, Jinlong Zhao, Naixin Lv, Anqing Fu, Chengxian Yin, Chengjun Song, Min Chao

**Affiliations:** 1 State Key Laboratory for Performance and Structure Safety of Petroleum Tubular Goods and Equipment Materials, CNPC Tubular Goods Research Institute, Xi’an 710077, China; lvnx@cnpc.com.cn (N.L.); fuanqing@cnpc.com.cn (A.F.); yincx@cnpc.com.cn (C.Y.); 2 School of Chemical Engineering and Technology, Xi’an Jiaotong University, Xi’an 710049, China; 3 Petrochina Jidong Oilfield Company, Tangshan 063004, China; yj_zhaojl@petrochina.com.cn; 4 Polymer Materials & Engineering Department, School of Materials Science & Engineering, Chang’an University, Xi’an 710064, China; spike725@163.com

**Keywords:** MXene, epoxy resin, amine curing agent, curing kinetics, molecular dynamic simulations, non-isothermal DSC

## Abstract

Herein, the curing kinetics and the glass transition temperature (*T_g_*) of MXene/phenolic epoxy composites with two curing agents, i.e., 4,4-diaminodiphenyl sulfone (DDS) and dicyandiamine (DICY), are systematically investigated using experimental characterization, mathematical modeling and molecular dynamics simulations. The effect of MXene content on an epoxy resin/amine curing agent system is also studied. These results reveal that the MXene/epoxy composites with both curing agent systems conform to the SB(m,n) two-parameter autocatalytic model. The addition of MXene accelerated the curing of the epoxy composite and increased the *T_g_* by about 20 K. In addition, molecular dynamics were used to simulate the *T_g_* of the cross-linked MXene/epoxy composites and to analyze microstructural features such as the free volume fraction (*FFV*). The simulation results show that the introduction of MXene improves the *T_g_* and *FFV* of the simulated system. This is because the introduction of MXene restricts the movement of the epoxy/curing agent system. The conclusions are in good agreement with the experimental results.

## 1. Introduction

Epoxy resins are widely used in aerospace [1], coatings [2], biomedical [3] and other fields because of their outstanding mechanical properties, chemical resistance and adhesive properties. As a thermosetting prepolymer, epoxy resin itself needs a crosslinking reaction with a curing agent to exert its properties. However, the crosslinking toughness with the curing agent is poor, the mechanical strength is not high, and the thermal performance is poor [4], which does not meet expectations. In addition, due to the wide variety of epoxy resins, some have potential but lack research; the curing characteristics are unclear, and their application and development are limited. Understanding the curing characteristics of epoxy and its performance enhancement are an ongoing research topics.

Since the curing of epoxy resin is often a complex and chaotic multi-step reaction, studying the curing kinetics of a particular epoxy resin can help us understand the mechanism of the physical and chemical processes of polymers by expressing the kinetic rates in the form of mathematical equations using temperature and other parameters [5]. Typically, thermal data are obtained by differential scanning calorimetry (DSC), which are processed to obtain kinetic properties. The feasibility of using DSC to study curing reactions has been verified [6] by Um et al. [7] and Rosu et al. [8], who investigated the kinetics of epoxy curing through isothermal and non-isothermal DSC methods. They both used the Málek method for the kinetic analysis of the data obtained by heat treatment and to validate the most consistent model for the curing reaction of epoxy resins using the Sesták–Berggren equation. Both of the experimental results exhibited excellent consistency with fitted predictions. These methods are still used today for helping to understand unknown epoxy curing processes [9,10].

Furthermore, certain properties of epoxy resins, such as curing degree, cross-linking density and glass transition temperature (*T_g_*), can significantly affect the formation process and heat resistance of epoxy resin [11]. To further investigate the relationship between the cross-linking process and the material properties of the epoxy systems, the main reliance has been on iterative experimental methods, which are both time-consuming and expensive. Currently, molecular dynamic (MD) simulations have become a particularly valuable tool to guide experiments, and also predict material properties [12]. For instance, Fan et al. [13] constructed periodic amorphous structures of cross-linked epoxy compounds using MD simulations to estimate properties such as the *T_g_*, coefficient of thermal expansion and Young’s modulus of the cross-linked epoxy compounds. Carla E. Estridge [14] used MD to investigate the effect of chain motion on properties such as *T_g_* during the curing of epoxy resins. Mohammad et al. [15] have employed experiment, model calculation, and MD simulation to study the curing kinetics of epoxy composites. The predicted values of these material properties are in good agreement with the experimental values in the literature. Hence, the combination of MD simulation and curing kinetics will become a mainstream method to study the curing characteristics and properties of epoxy resin.

According to available studies, the physical and chemical properties of epoxy resins are adjusted by changing the proportion of epoxy resin and curing agent [16], regulating the processes [17] and adding second phase fillers [18] to meet specific requirements. Carbon nanomaterials, such as graphene and carbon nanotubes, are often used as fillers in epoxy systems to improve thermal and mechanical properties, while MXene, an emerging two-dimensional nanomaterial, has gradually penetrated the field of epoxy resins as a research hotspot in recent years [19]. MXene has both ceramic and metallic properties, including high strength and modulus, chemical stability, excellent electrical and thermal conductivity and good processability [20]. The most common member of the MXene family, i.e., laminated Ti_3_C_2_T_x_, where *T* and *_x_* stand for different types of functional groups and their number, was prepared by chemically etching the Al layer from the parent MAX phase, i.e., Ti_3_AlC_2_ [21]. MXene requires few pretreatments because Ti lies on the MXene surface, which can easily bond with O atoms in EP resins [22]. Therefore, MXene can be well-dispersed in polymeric resins, resulting in highly conductive MXene/epoxy composites using simple processing methods [23]. MXene is a viable epoxy-resin reinforced phase. Current studies based on MXene/epoxy focus on the thermal and mechanical properties of the composites [24]. Liu et al. [25] prepared MXene functionalized epoxy composites and tested the tensile and flexural strengths of the materials. Sliozberg et al. [26] adopted the research method of combining the experimental test and molecular simulation for MXene/epoxy resin composites. Wang et al. [27] by adding nano silver/MXene multi-dimensional filler into epoxy found that the thermal conductivity of the composite was greatly improved.

Recently, several studies [28,29,30,31] have demonstrated that the curing kinetics method of epoxy nanocomposites are quite mature. Farhad et al. [32] used non-isothermal DSC curing kinetics to evaluate the curing response of graphene oxide/epoxy nanomaterials. Liu et al. [33] also used a similar approach to investigate the effect of various inorganic fillers on the curing kinetics of bisphenol A epoxy resin/polyamide systems under isothermal curing conditions. Meanwhile, MD simulation has also been widely used in the study of nanomaterials [34]. Marzieh et al. [35] conducted a simulation study of the thermomechanical properties of ZnMoO_4_ nanoparticles and epoxy resin systems. Wang et al. [36] used MD simulation to study the thermal conductivity of epoxy/MXene and verified that Ti_3_C_2_O_2_ MXene can significantly improve it. Ti_3_C_2_T_x_ MXene is a very promising epoxy-resin reinforced-phase material currently [23]; however, the curing process is still unclear, and few studies have applied MD techniques to the crucial study of the curing kinetics of MXene/epoxy resin systems.

Herein, PNE-177 phenolic epoxy resin and two common amine curing agents, i.e., 4,4-diaminodiphenyl sulfone (DDS) and dicyandiamine (DICY), were selected as reactants. A combination of MD simulations and experimental characterization was applied to obtain the curing kinetic parameters and *T_g_* of MXene/epoxy nanocomposites. The current work aimed to investigate the influence of MXene fillers on the curing behavior and thermomechanical properties of epoxy resins from macroscopic and microscopic perspectives. First, the non-isothermal DSC analysis was carried out to obtain the characteristic curing temperature of the MXene/epoxy system under different amine curing agents and determine the curing process. The experimental methods, such as Fourier transform infrared (FTIR) spectroscopy and isothermal DSC, were also utilized to obtain the curing degree, *T_g_* and relevant thermal parameters. Then, the curing kinetics of the system were investigated and a series of kinetic parameters were obtained using both model-free and model-fitting systems. In addition, the current work utilized MD simulations to supervise primary chemical and structural transformations during the growth of cross-linked networks. Furthermore, the free volume was also calculated using MD simulations. *T_g_* values of these systems were simulated to obtain a correlation between microstructure and macroscopic features. Finally, the results of curing kinetics, model fitting and MD simulations were compared with the experimental results of the MXene/epoxy nanocomposites. It was confirmed that Ti_3_C_2_T_x_ MXene can improve the *T_g_* of epoxy resin and promote curing.

## 2. Experimental

### 2.1. Materials

Phenolic epoxy resin (PNE-177) was obtained from Tiantai High-Tech Co. Ltd., Guangzhou, China, with an epoxy equivalent of 175–190 g/eq. 4,4′-diaminodiphenyl sulfone (DDS, Aladdin Industrial Co. Ltd., Shanghai, China, molecular weight: 248.30) and dicyandiamine (DICY, Shanghai Aladdin Industrial Co. Ltd., Shanghai, China, molecular weight: 84.08) were used as curing agents. Titanium aluminum carbide (Ti_3_AlC_2_, 2400 mesh, 98%) was purchased from Jilin Yiyi technology (Changchun, China). Lithium fluoride (LIF, AR,99%) was purchased from Shanghai Aladdin Chemical Reagent Co., Ltd. (Shanghai, China) Ti_3_C_2_T_x_ MXene was prepared in the laboratory. The solvents were acetone and N,N-dimethylformamide.

### 2.2. Preparation of MXene/Epoxy Resin Nanocomposites

**Figure 1:**
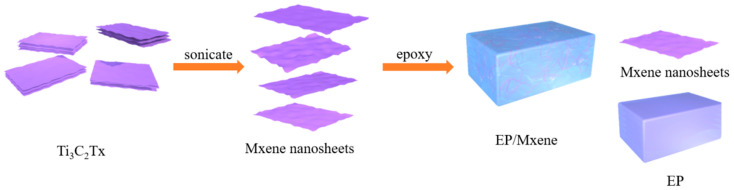
The flow chart of MXene/epoxy resin preparation.

MXene nanosheets were prepared according to the methods previously reported in the laboratory [37]. MXene/epoxy resin nanocomposites were prepared as follows: (a) dissolve a certain amount of MXene in 5 mL acetone, fully stir and apply ultrasound (40 kHz, 300 W) for 1 h to obtain the suspension; (b) pour it into PNE-177 epoxy resin which is heated and melted in advance, stir it evenly, and apply ultrasound (40 kHz, 300 W) at 80 °C for 2 h to fully disperse it; (c) after acetone volatilizes, add the calculated curing agent, wherein dicy is predissolved in 2 mL of N,N-dimethylformamide, heated and stirred for 30 min, and the solvent volatilizes at 90 °C for 2 h under vacuum; DDS is added into the epoxy resin, preheated to 110 °C and fully stirred for 30 min; (d) naturally cool the samples subject to non-isothermal DSC test and store in the refrigerator for freezing for standby. For samples subject to infrared and *T_g_* test pour onto a tetrafluoro plate (50 mm × 20 mm × 5 mm), and cure in the oven. The curing process is obtained from the characteristic temperature extrapolated by non-isothermal DSC. The PNE-177/dds curing process is 180 °C 1 h + 150 °C 2 h, and the PNE-177/dicy curing process is 150 °C 3 h. In the experiment, the content of MXene was 1, 3, 5 wt. %, respectively. Figure 1 shows the flow chart of MXene/epoxy resin preparation. In order to facilitate comparison, pure epoxy materials were prepared by similar methods.

### 2.3. Material Characterization

The s-4800 scanning electron microscope was used to observe the micro morphology of MXene. Fourier transform infrared spectroscopy was performed on a sensor II instrument, scanning from 4000–400 cm^−1^ using attenuated total reflection mode. XRD was tested on d8-advance instrument. A SDT-650 synchronous thermal analyzer was used to characfiguterize the thermal stability of the samples. The approximate curing degree of the epoxy composites was analyzed by Fourier transform infrared spectroscopy (FTIR) and differential scanning calorimetry (DSC). The non-isothermal DSC was used to obtain the characteristic curing temperature and reaction rate. The test conditions were as follows: a small amount of sample was sealed in an alumina crucible and heated from RT to 300 °C under N_2_ atmosphere through different heating rates (5 °C/min, 10 °C/min, 15 °C/min and 20 °C/min). The *T_g_* of the cured sample was obtained by isothermal DSC, and the test conditions were as follows: the specimens were heated from *RT* to 300 °C under N_2_ at a heating rate of 5 °C/min.

### 2.4. Curing Kinetics

The physical properties of epoxy resins strongly depend on their structure, curing degree, curing conditions, curing time and temperature [38]. Therefore, it is necessary to investigate the relationship between the curing behavior and final properties to obtain a high-performance resin.

Non-isothermal DSC is often used as a valuable tool to study the curing kinetics of epoxy composites. In general, a small amount of sample is sealed in an alumina crucible and heated from *RT* to 300 °C under N_2_ atmosphere at different ramping rates (5 °C/min, 10 °C/min, 15 °C/min and 20 °C/min). It is considered that the exotherm at a certain moment is proportional to the total degree of reaction of the reacting functional groups [39]. Therefore, the degree of reaction (*α*) at a certain moment can be defined as Equation (1):

(1) $$\alpha =\frac{H_{\alpha }}{\Delta H}$$

where *H_α_* refers to the exothermic enthalpy of the curing reaction, which can be calculated by integrating the exothermic peak of the sample at a given moment in time, and Δ*H* represents the total exothermic enthalpy of the curing reaction, i.e., the integral of the total exotherm. The basic assumption in the study of thermoset polymer curing processes using DSC is that the reaction rate (*dα*/*dt*) is directly proportional to the heat flow (Φ) [40], as in Equation (2).

(2) $$\frac{d\alpha }{dt}=\frac{\Phi }{\Delta H}$$

The rate of the curing reaction can also be described as a function both with temperature (*T*) and conversion rate (*α*), as given in Equation (3).

(3) $$\frac{d\alpha }{dt}=k\left(T\right)f\left(\alpha \right)$$

where, *T* refers to the time and *f*(*α*) represents the reaction model depending on the type of curing agent and by-product reaction, which will be described later in the calculations of *k*(*T*). The reaction rate constant can be expressed in terms of the Arrhenius model (Equation (4)).

(4) $$k\left(T\right)=Aexp\left(-\frac{E_{a}}{RT}\right)$$

where *A* refers to a pre-factor, *E_a_* represents the apparent activation energy and *R* denotes the universal gas constant. When combined with the Arrhenius equation, the curing rate can be described by Equation (5):

(5) $$\frac{d\alpha }{dt}=Aexp\left(-\frac{E_{a}}{RT}\right)f\left(\alpha \right)$$

At this point, a preliminary calculation model of curing kinetics of the epoxy composites can be obtained.

Next, model-free and model-fitting methods can be used to obtain reliable and consistent kinetics information [41]. The commonly used non-model kinetic methods, such as the Flynn–Wall–Ozawa method (FWO) [42,43] and Kissinger–Akahira–Sunose method (KAS) [44], are adopted to analyze the curing kinetics of epoxy resin systems. Herein, both methods were used to calculate and compare the kinetics parameters of the epoxy/curing agent system, and the KAS function (Equation (6)) gives the relationship between reaction activation energy (*E_a_*) and peak temperature (*T_p_*):

(6) $$\ln\left(\frac{\beta }{T_{p}^{2}}\right)=\ln\left(\frac{AR}{\Delta E_{a}}\right)-\frac{E_{a}}{RT_{p}}$$

where *β* refers to the warming rate. Furthermore, as one of the model-free iso-conversion rate methods, FWO provides a simple relationship between *E_a_* on α and the associated conversion rate temperature (*T_α_*) (Equation (7)):

(7) $$ln\beta =const-\frac{1.052E_{a}}{RT_{\alpha }}$$

Since the *E_a_* is determined, selecting a kinetics model that can describe the solidification data is possible. The model-fitting methods tend to receive highly uncertain values of Arrhenius parameters, and the use of iso-conversion methods can avoid the above disadvantages of non-model fitting [45]. The most suitable kinetics model can be chosen according to the Málek iso-transformation method [41], which allows us to calculate a comprehensive set of significative kinetic parameters to represent the solidification reaction. Málek’s method has two eigenfunctions, i.e., *y*(*α*) and *z*(*α*), which can be defined as Equations (8) and (9):

(8) y(α)=(dα/dt)exp(x)

(9) z(α)=π(x)(dα/dt)(T/β)

where *x* = *E_a_*/*RT*, *β* refers to the warming rate (K min^−1^) and *T* represents the absolute temperature (K). As mentioned earlier, *π*(*x*) function can be approximated through the 4th rational expression of Senum and Yang [46], as given in Equation (10):

(10) $$\left(x\right)=\frac{x^{3}+19x^{2}+88x+96}{x^{4}+20x^{3}+120x^{2}+240x+120}$$

### 2.5. Determination of Curing Degree

The curing degree is an essential precondition for researching the properties of thermoset polymeric materials [39]. FT-IR is a commonly used characterization method to measure the conversion rate of epoxy resin/curing agent systems. Herein, after performing non-isothermal DSC analysis of the epoxy resin to determine the curing temperature, we compared the changes in the intensity of the epoxy groups before and after curing in FT-IR spectra, which were used to measure the degree of curing.

## 3. MD Simulations

**Figure 2:**
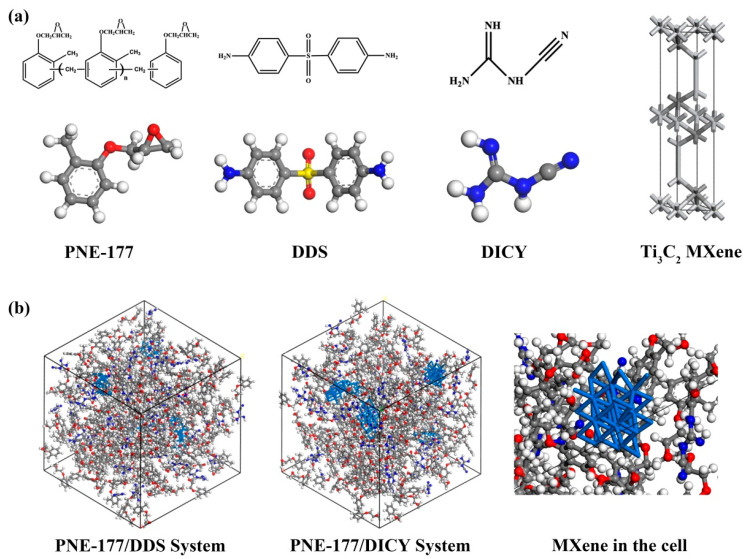
The molecular structure (**a**) and epoxy resin system (**b**) using MD simulations.

Herein, Material Studio 2017 software was used to build the reactant molecules and simulate the curing process. The chemical structures of the phenolic epoxy resin (PNE-177), curing agents, i.e., 4,4-diaminodiphenyl sulfone (DDS) and dicyandiamine (DICY), and Ti_3_C_2_ MXene are shown in Figure 2a using the Visualizer module. The pure epoxy/curing agent system and MXene-containing nanocomposites were constructed by the Amorphous Cell module, as shown in Figure 2b. The mass ratio of epoxy to curing agent was PNE-177:DDS:DICY = 100:35.02:11.86, which was calculated based on the number of amine groups and epoxy equivalents. Then, 1, 3 and 5 wt. % of MXene were added with the size of 2 Å × 2 Å. The epoxy resin system was equivalent to more than 8000~9000 atoms, and the model size of the unit cell was 50 × 50 × 50 Å^3^, which rendered excellent reliability [47].

Before the construction of the Amorphous Cell, all molecular models were performed using the Forcite module for energy minimization; the charge of the reacting atoms was set reasonably to maintain the system charge neutral. Two different force fields were used to simulate the intramolecular interactions of the pure epoxy and MXene/epoxy systems because of the presence of *Ti* atoms in the composite. Thus, the COMPASS II force field was chosen to predict the pure polymeric material [48] and the Universal force field for the composites. In addition, a Nose–Hoover thermostat was used to regulate the monitoring of temperature and a Berendsen barostat was used to regulate the pressure, as well as van der Waals interactions and Coulomb potential were calculated by using the atom-based method and Ewald method, respectively. The real space cutoff for nonbonded interactions was set at 9.5 Å with a buffer of 0.5 Å.

The cross-linking process was also simulated. First, the reaction site atoms were set between reactants and the O reaction sites generated hydroxyl groups, leaving the C reaction sites. Then, the presence of NH_3_ in the curing agent molecule was dehydrogenated to provide the N reactive sites. This procedure identified the reactive sites within a 10 Å cutoff to form chemical bonds. Then, the optimization was performed and a cross-linking network was obtained, and the aforementioned steps were repeated until no reactive atoms were present within the reaction distance or the degree of cure reached a preset value, the curing degree of this experiment was determined to be 85% based on the above infrared test results.

## 4. Results and Discussion

### 4.1. Characterization of MXene Nanosheets

MXene nanosheets were obtained by the selective etching of the Al atomic layer in Ti_3_AlC_2_ with hydrochloric acid and lithium fluoride. The SEM, FTIR, XRD and TGA performance tests results of MXene are shown in Figure S1. Figure S1a is the SEM image of the MXene nanosheets. It can be seen that the MXene nanosheets have a sheet-like structure, with a size of approx. 200 μm. Fourier transform infrared spectroscopy can also prove the successful synthesis of MXene nanosheets (Figure S1b). The wide absorption peak at about 3448 cm^−1^ belongs to -OH, with the Ti-O absorption peak belonging to MXene at 669 cm^−1^. In addition, in the XRD curve, the diffraction peak at 39.1° in Ti_3_AlC_2_ disappeared in MXene, and the (002) diffraction peak in XRD moved from 9.39° to 6.67°. This was due to the chemical etching of the Al atomic layer [49]. It can be seen from the thermogravimetric images that the residual weight of the MXene nanosheet at 800 °C was 85.92%, which has good thermal stability. In summary, the results show that by etching the Al atomic layer in Max, a few layered MXene nanosheets were successfully prepared.

### 4.2. Curing Kinetics

#### Non-Isothermal DSC Analysis

**Figure 3:**
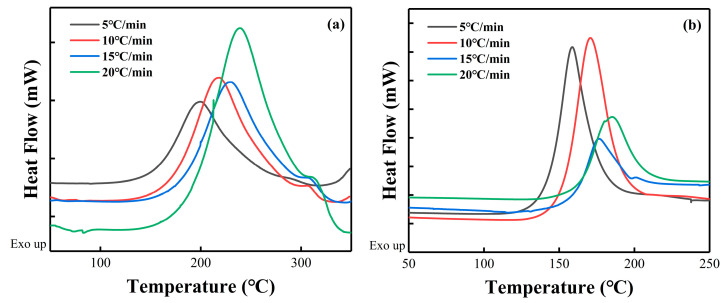
The non-isothermal DSC curves of (**a**) PNE-177/DDS and (**b**) PNE-177/DICY.

The non-isothermal DSC curves of the pure PNE-177/DDS and PNE-177/DICY systems are presented in Figure 3, showing the curing exothermic peaks at different heating rates. Table 1 summarizes the curing characteristic temperature parameters, such as onset, peak and termination temperatures (*T_i_*, *T_p_* and *T_f_*), of the curing process of both of the epoxy/curing agent systems at different ramp rates. It can be observed that the *T_i_*, *T_P_* and *T_f_* of the two systems move towards high temperature with the increase of the heating rate, indicating that the curing reaction is a dynamic process [50]. At a lower heating rate, the system has enough time to react, and the starting temperature is low. With the increase of reaction rate, thermal effect and thermal inertia, the characteristic temperature moves backward [51].

Table 2 presents the linear fitting of the characteristic temperatures according to different ramp rates for different MXene contents, resulting in the extrapolated characteristic temperatures for each system. These parameters can be used as a reference for selecting the best curing process during actual experiments [52], and Figure S2 shows the linear fitting curves of the characteristic temperature for pure PNE-177/DDS and PNE-177/DICY systems. The curing temperature (*T_cure_*) was determined at *T_i_* < *T_cure_*< *T_p_*. Therefore, the curing conditions of PNE-177/DDS and PNE-177/DICY were 180 °C and 150 °C, respectively. In addition, it can be seen that the addition of MXene affects the characteristic curing temperature of epoxy. For instance, the increase in MXene content increases the *T_i_* and *T_p_* values. This is because the addition of fillers increases the reaction energy barrier in the initial stage of the system [53], making the reaction slower at the beginning. However, the increase of the *T_f_* value implies that the -OH on the surface of MXene participates in the curing reaction of the epoxy resin [54]. Fillers increase the reactivity of the system, shorten the cure time, and increase the time to reach the final temperature [33].

### 4.3. Reaction Activation Energy and Conversion Rate

Miller et al. [55] reported that the apparent activation energy (*E_a_*) could be used to represent the energy barrier and reflect the process of the curing reaction. Because the *E_a_* of epoxy resin is not a constant [56], it is a function of the transformation during reaction, the study of *E_a_* helps us to understand the curing process. Herein, we used the FWO method to study the reaction activation energy of several experimental systems, and selected a set of *α* from a series of experimental results with a step size of 0.05 (*α* = 0.05, 0.10, …, 0.95). The *E_a_* of the curing reaction was calculated using Origin2017 and EXCEL software, and plotted against the conversion rate (*α*).

**Figure 4:**
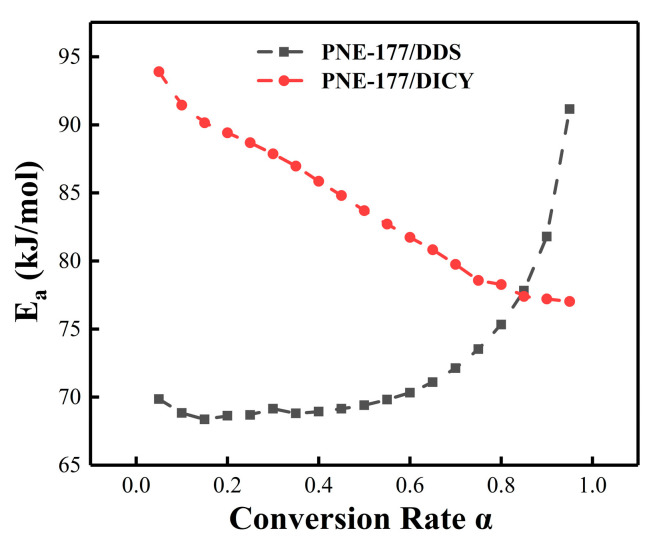
*E_a_* vs. *α* curves of different curing agent systems.

The activation energy and conversion rate relationship curves could be obtained according to the trend, showing that *E_a_* continuously changed with the increase of *α* and exhibited different characteristics for both PNE-177/DDS and PNE-177/DICY systems. In the pure epoxy/curing agent system (Figure 4), the *E_a_* of PNE-177/DDS was low and relatively smooth at the beginning (*α* = 0.05–0.55) and gradually increased with the reaction procession. This means that the initial reactivity of the system was high and the reaction required less energy to proceed. Moreover, as the curing reaction proceeded, the resin underwent gelation or a curing reaction and the movement of polymer molecular chains was gradually restricted [57]. When the reaction was further deepened (*α* > 0.80), the chain movement required high energy from outside and it was difficult for the reaction to proceed completely; the activation energy of the reaction gradually increased. The overall activation energy of the PNE-177/DICY system was significantly higher than the DDS curing agent system, which was related to the fact that DICY is a latent curing agent [58]. This curing agent can be mixed with an epoxy system to enhance the stability for a long time, which means that the reaction at the beginning of the energy potential is high. However, unlike PNE-177/DDS, the *E_a_* of this system decreased significantly as the conversion proceeded, indicating that the system reached a certain conversion rate where the reactants were more likely to collide, leading to increased chain segment activity and a low energy barrier for the curing reaction [59].

**Figure 5:**
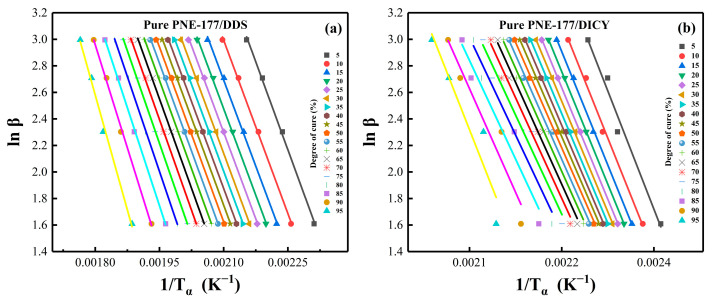
The linear fitting via FWO method with different MXene content: (**a**) PNE-177/DDS and (**b**) PNE-177/DICY.

**Figure 6:**
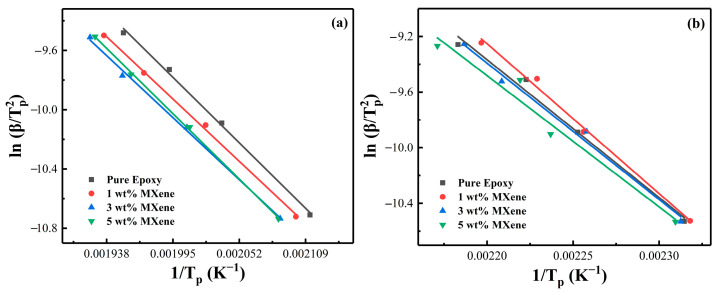
The linear fitting via KAS method with different MXene content: (**a**) PNE-177/DDS and (**b**) PNE-177/DICY.

**Figure 7:**
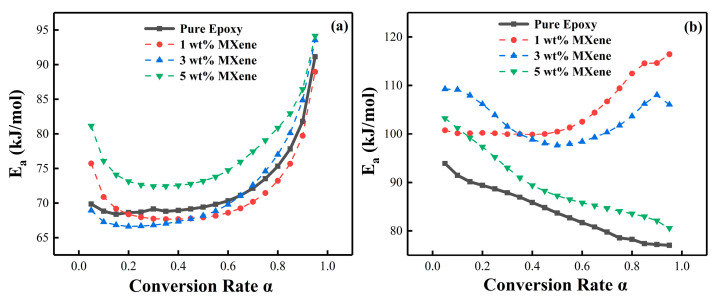
The relationship between different MXene content, *E_a_* and *α*: (**a**) PNE-177/DDS and (**b**) PNE-177/DICY.

The effect of different amounts of MXene loading on the activation energy of the epoxy/curing agent system was also investigated. Using the FWO method (Figure 5) and the KAS method (Figure 6) to linearly fit the data, we discuss the *E_a_* of epoxy together to compare the difference in activation energy obtained by different fitting methods for the same system. Figure 7 shows the relationship between the activation energy and the conversion rate of the two systems with different MXene content. It can be seen from the trend in Figure 7a that the overall activation energy of MXene was lower than that of pure epoxy when the content of MXene was 1 wt. % and 3 wt. %, and it was significantly increased when the content was 5 wt. %. The incorporation of a small amount of MXene (1 wt. %, 3 wt. %) participated in the crosslinking reaction [54] and slightly reduced the activation energy of the system, but the introduction of fillers greatly restricted the movement space of the polymer chains and monomers, hindering the initial reaction of the radicals and the movement between clusters [27,60,61], so the activation energy at the initial stage of the reaction increased, which was also the reason for the overall high activation energy of the system when the addition amount reached 5 wt. %. The increase of *E_a_* in the later stage of the reaction was because the increase of viscosity and the consumption of reactive groups made the curing reaction difficult as the reaction progressed [62].

Furthermore, Table 3 compares the activation energy calculated by fitting using the FWO and KAS methods. There are some differences between the two methods. In general, the activation energy calculated by the KAS method is more accurate and reliable, but it is difficult to involve the whole curing process, whereas the FWO method, although slightly less accurate, includes the whole curing process [57].

### 4.4. Málek Method Analysis

**Figure 8:**
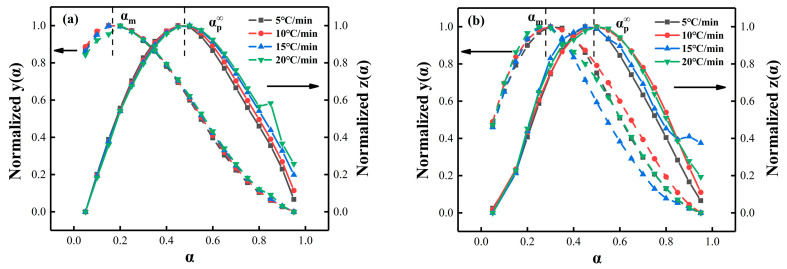
The normalized *y*(*α*) vs. *z*(*α*) curves of (**a**) PNE-177/DDS and (**b**) PNE-177/DICY systems.

Herein, the curing kinetics model of the MXene/epoxy composite system was also determined based on the Málek model. The average *E_a_* values, calculated through the FWO method, were first introduced into the *y*(*α*) and *z*(*α*) of Equations (8) and (9) to obtain the function curves, and the values needed to be normalized for simplicity. The obtained function plots are shown in Figure 8, taking the extreme value points (α_M_ and $\alpha_{p}^{\infty }$) of both fitted curves. The Málek criteria were set as 0 < α_M_ < $\alpha_{p}^{\infty }$ and $\alpha_{p}^{\infty }$ ≠ 0.632, where the eigenvalues were determined by the kinetics model. If these criteria were met, the two-parameter Sestak–Berggren model (SB(m, n)) [63] was considered suitable to study the curing reaction and cross-linking process of the epoxy resin, which can be represented by Equation (11):

(11) $$f\left(\alpha \right)=\alpha^{m}{\left(1-\alpha \right)}^{n}$$

(12) ln(dαdtexp(EaRT))=lnA+nln[αmn(1−α)]

(13) $$p=\frac{m}{n}=\frac{\alpha_{M}}{1-\alpha_{M}}$$

**Figure 9:**
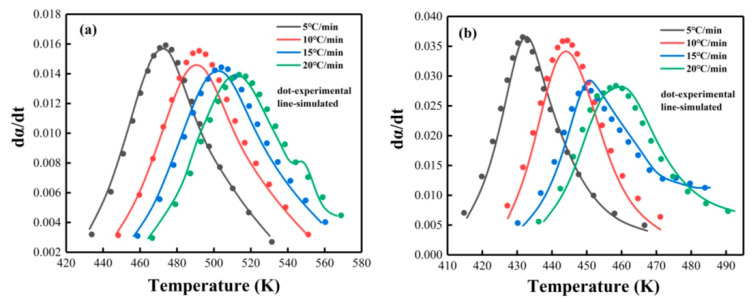
The comparison between experimental and simulated DSC curves of (**a**) PNE-177/DDS and (**b**) PNE-177/DICY systems based on SB (m, n) model.

The parameter *p* was first determined from the value of $\alpha_{M}$ (Equation (13)), then the logarithm was taken after substituting it into the rate equation (Equation (5)), *n* and ln*A* were then obtained from the slope and intercept of the linear correlation ln[y(α)] with $\ln\left[\alpha^{p}\left(1-\alpha \right)\right]$, respectively (Equation (12)). The parameter m was calculated by *m* = *p* × *n*. Then, the kinetic parameters was determined, and the average values were listed in Table S1. The rate equation can be obtained by the calculated kinetics parameters at present. Proposed with the SB (m, n) equation, the kinetics model was verified by comparing the experimental curve of *dα*/*dt* vs. *T* with the simulated curve using the data in Table S1; the comparison is shown in Figure 9. The excellent consistency between the simulated and experimental data indicates that the autocatalytic reaction model was suitable for describing the non-isothermal curing reaction process of PNE-177/DDS and PNE-177/DDS systems [64], and the addition of MXene did not affect the reaction model.

### 4.5. Curing Degree

**Figure 10:**
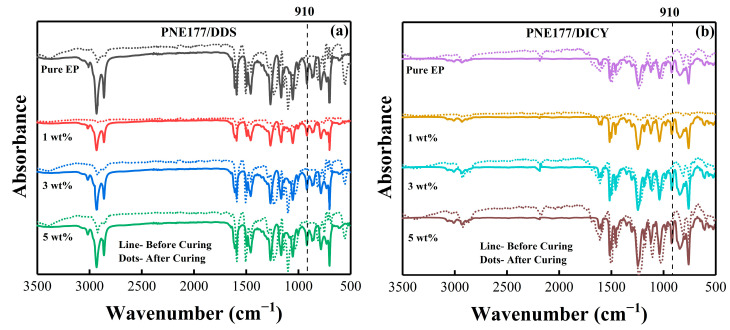
FTIR spectra of PNE-177/DDS (**a**) and PNE-177/DICY (**b**) system epoxy resin.

The final epoxy group curing degree can be obtained via FT-IR spectra as shown in Figure 10; the absorption peak of the epoxy group (about 910 cm^−1^) disappears after curing, which indicating that the epoxy group is completely involved in the cross-linking reaction between the PNE-177/DDS system and the PNE-177/DICY system during the curing process.

Meanwhile, the isothermal DSC technique monitors the heat flow within a sample passing through a specific heat distribution and is also used to measure the curing degree. The same DSC scans were performed on the epoxy resin before and after curing and the samples in this study were heated from RT to 300 °C at a heating rate of 5 °C/min. The test yields the final curing degree of the epoxy/hardener system after curing, which can be used as a reference for building conditions for the MD model. Equation (14) is a rough calculation of the curing degree of epoxy [59].

(14) $$X=\left(1-\frac{A_{1}}{A_{0}}\right)\times 100\%$$

where *A_0_* refers to the exothermic peak area before curing reaction and and *A_1_* implies the area after cure, respectively. Table S2 shows the exothermic peak areas and the calculated curing degree of the MXene/epoxy composites before and after curing at a heating rate of 5 °C/min. It can be seen that the epoxy composites of both curing agent systems were completely cured and the addition of MXene increased the curing degree. Hence, the curing degree of the epoxy was finally controlled to be around 85% in the subsequent simulation setup.

### 4.6. Glass Transition Temperature

**Figure 11:**
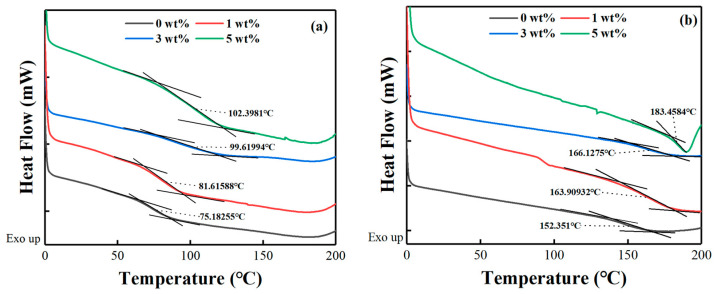
DSC curves of (**a**) PNE-177/DDS and (**b**) PNE-177/DICY systems at the heating rate of 5 °C/min.

The glass transition temperature (*T_g_*) is a key parameter that must be considered for thermal stability in polymeric applications. Figure 11 presents isothermal DSC plots of the cured epoxy for each system at a heating rate of 5 °C/min from RT to 300 °C. *T_g_* can be determined by the inflection point of the DSC curve [65]. It can be seen that the *T_g_* of the pure PNE-177/DDS system and PNE-177/DICY system was 75.18 °C and 152.3 °C, respectively. In each group of experiments, the addition of MXene significantly increased the *T_g_* of the epoxy/curing agent system. This is because the Ti_3_C_2_ MXene filler added to the epoxy resin matrix can be used as a physical interlock point, which is intertwined by the epoxy resin chain, which is conducive to limiting the epoxy resin chain and reducing their movement [66,67].

**Figure 12:**
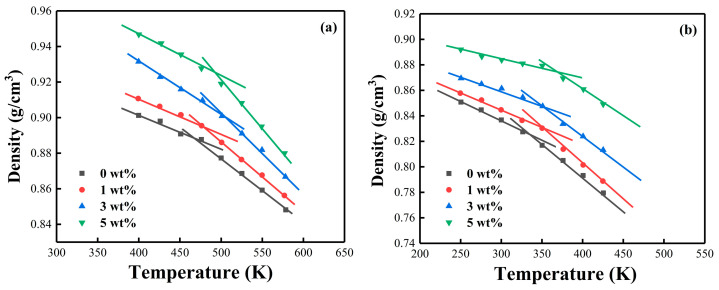
The density vs. temperature curves of MXene/epoxy resin based on MD simulations: (**a**) PNE-177/DDS and (**b**) PNE-177/DICY.

Several studies have used MD simulations to assess the key properties of the polymer, such as *T_g_* [68,69,70]. Referring to the previous work [71], the current study performed annealing simulations using an NPT ensemble from 600 to 300 K at 1 atm with a cooling rate of 20 K/500 ps, repeated 10 times and averaged the density at each temperature to create a curve fitting the density dependence on temperature. Then, the value of *T_g_* was obtained from the inflection point of the curve. Figure 12 presents the density vs. temperature plots of both cross-linked epoxy resin systems during cooling. The simulated values were slightly higher than the experimental values, but the trend remained the same, the *T_g_* of the PNE-177/DICY system was higher than the PNE-177/DDS system (Table 4). The higher simulated values were caused by the difference between simulated and experimental conditions, where the experimental heating rate was 5 °C/min and the cooling rate in the MD simulation environment was about 10^11^ times faster than the actual cooling rate because of the microscopic nature of MD simulations [72,73].

### 4.7. Free Volume

The free volume is an important factor that affects the thermal–mechanical properties of polymers. In the glassy state, it is proposed that the movement of molecular chains are inhibited by the reduction of the free volume, leading to a higher *T_g_* [74]. According to this theory, the volume of material (*V_T_*) consists of the free volume (*V_f_*) and the occupied volume (*V_o_*), as given below:

(15) $$V_{T}=V_{o}+V_{f}$$

Meanwhile, *FFV*, which is a description of the relative amount of the free volume, can be calculated from Equation (16):

(16) $$FFV=\frac{V_{f}}{V_{o}+V_{f}}\times 100\%$$

**Figure 13:**
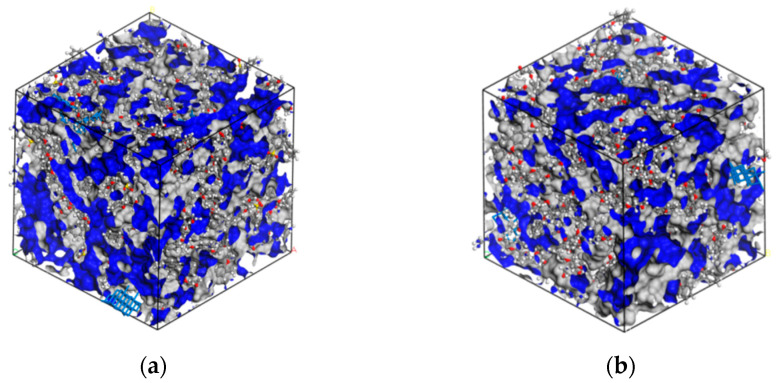
The image of free volume of unit cells: (**a**) PNE-177/DDS and (**b**) PNE-177/DICY systems.

The thermal mechanical properties can now be predicted via *FFV*. Herein, the free volume was obtained by calculating the Connolly Surface using the Atom Volume and Surface module, as shown in Table 5. Figure 13 shows the free volume model for the PNE-177/DDS and PNE-177/DICY systems, the grey part represents *V_o_* and the blue part represents *V_f_*. It was found that the FFV of the PNE-177/DDS system was higher than the PNE-177/DICY system, and the PNE-177/DICY system was more compact. Hence, it can be predicted that the *T_g_* of PNE-177/DICY should be higher than the PNE-177/DDS. Meanwhile, the introduction of MXene restricted the movement of chain segments of the epoxy/curing agent system, which reduced the free volume [75] and resulted in a higher *T_g_*. All these observations are consistent with the MD simulation and experimental results.

## 5. Conclusions

In summary, a combination of theoretical calculations and experimental characterization is employed to study the influence of two curing agents, i.e., PNE-177/DDS and PNE-177/DICY, and MXene content on curing kinetics and the *T_g_* of the MXene/epoxy composites. The following conclusions can be drawn from the current results.

First, the optimal curing temperature for the epoxy resin composites, with different MXene content, was determined using the non-isothermal DSC analysis. Then, the curing process and curing degree were analyzed using FIIR based on the optimal curing temperature. The results revealed that the optimal curing temperature of PNE-177/DDS system was 180 °C and PNE-177/DICY was 150 °C. The MXene/epoxy composites could achieve about 90% curing and the curing degree of the epoxy was improved after MXene addition. The *E_a_* calculated by the FWO and KAS methods demonstrated that the *E_a_* of the PNE-177/DDS system increased as the reaction proceeded and hindered the reaction occurrence, whereas the activation energy of the PNE-177/DICY system decreased with the increase in conversion rate. In addition, the addition of filler and concentration played a crucial role in the curing kinetics of the epoxy resin. For the PNE-177/DDS system, a small amount of MXene filling slightly reduced the activation energy of the system. In the presence of excessive MXene (5 wt. %), the reaction activation energy was significantly increased. The addition of MXene also increased the activation energy of the PNE-177/DICY system. Next, the results of model fitting using Málek et al.’s conversion rate method revealed that the curing mechanism of the MXene/epoxy composites, with DDS and DICY as curing agents, were complex and followed the SB (m, n) two-parameter autocatalytic model, rendering an excellent consistency with experimental results.

Moreover, the MXene/epoxy composites, with different curing agents and MXene content, were modeled using MD simulations. The cross-linking process was simulated based on the simulation and *T_g_*. The MD simulation results revealed that the *T_g_* of the epoxy system significantly increased by about 20K after MXene addition. Furthermore, the FFV in the free volume simulations also predicted the improvement of the thermomechanical properties of the MXene/epoxy composites.

Hence, the utilization of MXene, as a filler, modified the curing behavior and *T_g_* of the epoxy-based composites. The filler content rendered a significant influence on the reaction rate and reaction kinetics, which should be considered in practical applications of MXene/epoxy composites.

## Figures and Tables

**Table 1:** The characteristic temperatures of the pure epoxy/curing agent system.

Epoxy System	Β (°C/min)	*T_i_* (°C)	*T_p_* (°C)	*T_f_* (°C)
PNE-177/DDS	5	114.55	200.16	285.77
	10	120.01	217.77	298.77
	15	124.47	228.87	300.25
	20	129.00	239.03	306.01
PNE-177/DICY	5	113.8	158.89	204.77
	10	121.88	170.77	207.51
	5	125.44	176.75	215.31
	20	131.83	184.91	224.61

**Table 2:** The extrapolated characteristic temperatures of different MXene-doped PNE-177/DDS and PNE-177/DICY systems.

Epoxy System	MXene (wt%)	*T_i_* (°C)	*T_p_* (°C)	*T_f_* (°C)
PNE-177/DDS	0	110.05	189.53	282.15
	1	121.62	191.73	286.91
	3	122.875	195.2	276.05
	5	129.72	195.8	271.25
PNE-177/DICY	0	108.83	151.82	196.22
	1	111.99	152.27	196.64
	3	118.04	152.07	192.87
	5	117.7	153.08	191.85

**Table 3:** The activation energy of PNE-177/DDS and PNE-177/DICY systems based on both fitting methods.

Epoxy System	MXene (wt%)	FWO *E_a_* (kJ/mol)	KAS *E_a_* (kJ/mol)
PNE-177/DDS	0	72.25	64.40
	1	71.37	61.28
	3	71.88	61.02
	5	77.14	64.29
PNE-177/DICY	0	85.14	82.33
	1	104.42	89.51
	3	102.85	81.47
	5	89.49	78.42

**Table 4:** The experimentally measured and MD simulated *T_g_* values of PNE-177/DDS and PNE-177/DICY systems.

Epoxy System	MXene (wt%)	Experimental *T_g_* (K)	Simulation *T_g_* (K)
PNE-177/DDS	0	348.33	366.67
	1	354.27	375.31
	3	372.77	389.12
	5	375.54	396.08
PNE-177/DICY	0	425.50	437.27
	1	437.06	449.58
	3	439.28	452.09
	5	456.61	471.34

**Table 5:** The free volume parameters of PNE-177/DDS and PNE-177/DICY systems.

Epoxy System	MXene (wt%)	V_f_ (Å^3^)	V_T_ (Å^3^)	FFV (%)
PNE-177/DDS	0	2578.3	12,710.9	20.28
	1	2331.88	12,797.6	18.22
	3	2207.69	12,840.4	17.19
	5	2134.34	12,894.5	16.55
PNE-177/DICY	0	2112.54	11,548.2	18.29
	1	1899.41	11,625.8	16.34
	3	1802.55	11,709.9	15.39
	5	1743.09	11,774.3	14.80

## Data Availability

Not applicable.

## Supplementary Materials

The following supporting information can be downloaded at: https://www.mdpi.com/article/10.3390/nano12132249/s1, Figure S1: The illustration of synthesis of few layers Ti3C2Tx MXene; Figure S2: The characteristic fitting curves of (a) PNE-177/DDS and (b) PNE-177/DICY; Table S1: The Kinetics parameters of PNE-177/DDS and PNE-177/DICY systems based on non-isothermal DSC analysis.; Table S2: The exothermic peak area and curing degree of MXene/epoxy composite before and after curing at a heating rate of 5 °C/min.
