# Supplementary material for: Curing and Molecular Dynamics Simulation of MXene/Phenolic Epoxy Composites with Different Amine Curing Agent Systems
Source: Nanomaterials (Basel). 2022 Jun 30;12(13):2249. doi: 10.3390/nano12132249 (PMC9268527; doi:10.3390/nano12132249)
Supplement: Supplementary file 1 [file nanomaterials-12-02249-s001.zip › nanomaterials-1753373-supplementary.pdf]

Supporting Information for

Curing and molecular dynamics simulation of MXene/ phenolic epoxy composites with different amine curing agent systems

Rui Cai [1,2,*], Jinlong Zhao [3], Naixin Lv [1], Anqing Fu [1], Chengxian Yin [1], Chengjun Song [4] and Min Chao [4,*]

[1] State Key Laboratory for Performance and Structure Safety of Petroleum Tubular Goods and Equipment Materials, CNPC Tubular Goods Research Institute, Xi'an 710077, China; lvnx@cnpc.com.cn (N.L.); fuanqing@cnpc.com.cn (A.F.); yincx@cnpc.com.cn (C.Y.)

[2] School of Chemical Engineering and Technology, Xi'an Jiaotong University, Xi'an 710049, China

[3] Petrochina Jidong Oilfield Company, Tangshan 063004, China; yj_zhaojl@petrochina.com.cn

[4] Polymer Materials & Engineering Department, School of Materials Science & Engineering,
Chang'an University, Xi'an 710064, China; spike725@163.com

* Correspondence: cair@cnpc.com.cn (R.C.); chaomin@chd.edu.cn (M.C.)

Table of Contents

Figure S1. The illustration of synthesis of few layers $Ti_3C_2T_x$ MXene.

Figure S2. The characteristic fitting curves of (a) PNE-177/DDS and (b) PNE-177/DICY.

Table S1: The Kinetics parameters of PNE-177/DDS and PNE-177/DICY systems based on non-isothermal DSC analysis.

Table S2: The exothermic peak area and curing degree of MXene/epoxy composite before and after curing at a heating rate of 5 °C/min.

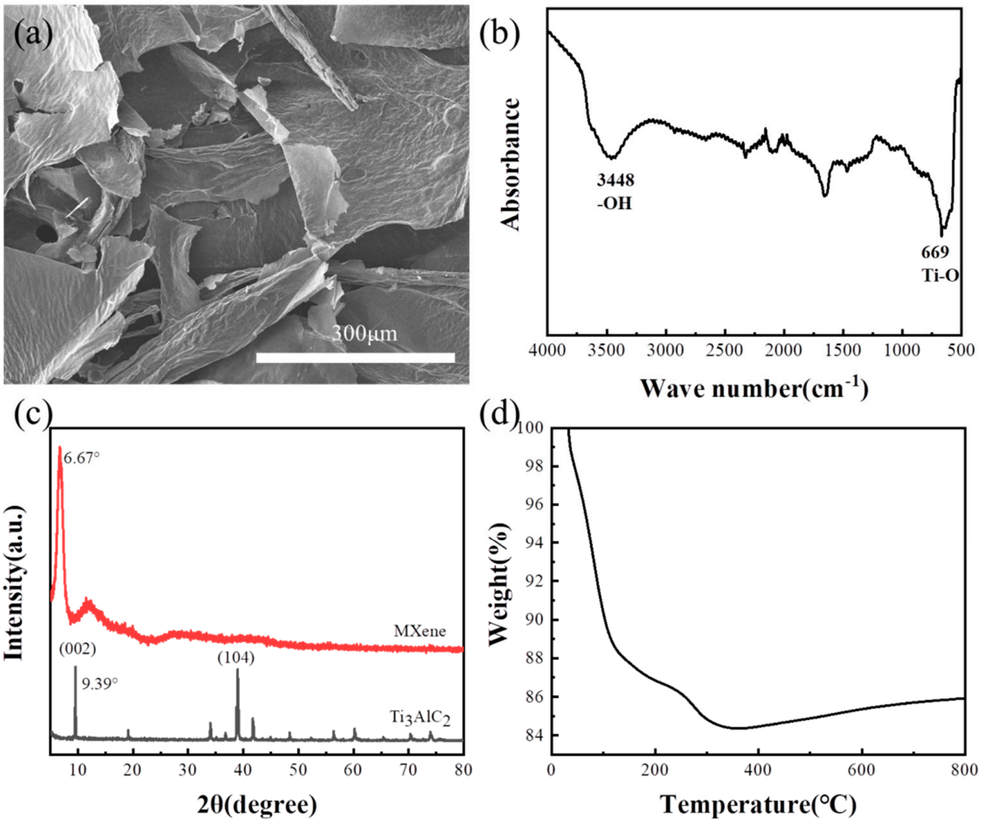

Figure S1 SEM (a), FTIR (b), XRD (c), and TGA (d) performance tests of MXene.

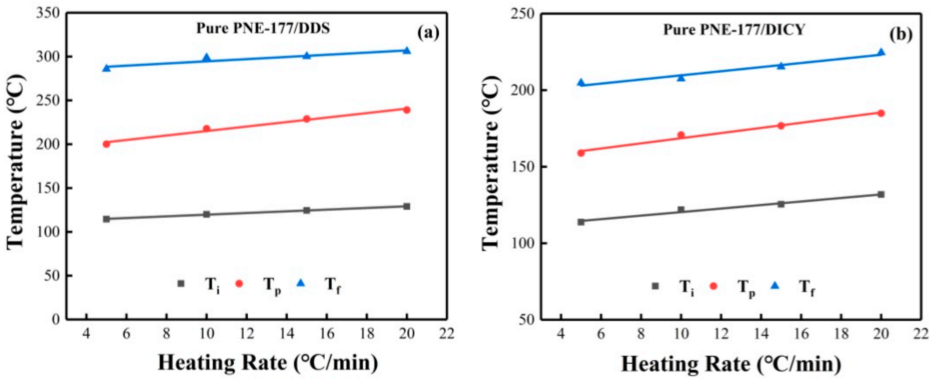

Figure S2. The characteristic fitting curves of (a) PNE-177/DDS and (b) PNE-177/DICY.

Table S1: The Kinetics parameters of PNE-177/DDS and PNE-177/DICY systems based on non-isothermal DSC analysis.

Epoxy System	MXene (wt%)	α_p	α_M	α_p^{∞}	p	m	n	lnA
PNE-	0	0.68	0.188	0.542	0.231	0.184	0.891	10.77
177/DDS	1	0.67	0.185	0.545	0.226	0.189	0.89	10.78
	3	0.66	0.184	0.54	0.225	0.191	0.887	10.74
	5	0.7	0.191	0.553	0.236	0.197	0.895	10.81
PNE-	0	0.48	0.315	0.58	0.459	0.553	1.441	11.09
177/DICY	1	0.47	0.322	0.587	0.474	0.556	1.439	11.11
	3	0.47	0.328	0.596	0.488	0.565	1.434	11.25
	5	0.46	0.331	0.599	0.494	0.57	1.446	11.29

Table S2: The exothermic peak area and curing degree of MXene/epoxy composite before and after curing at a heating rate of 5 ℃/min.

Epoxy System	MXene (wt%)	A_0	A_1	Curing degree(%)
PNE-177/DDS	0	444.012	52.839	89.1
	1	339.993	24.138	92.9
	3	360.379	23.424	93.5
	5	235.941	21.707	90.8
PNE-177/DICY	0	795.737	116.178	85.4
	1	349.628	34.613	90.1
	3	238.043	29.279	87.7
	5	351.83	37.998	89.2
